# Abdominal and Pelvic Organ Failure Induced by Intraperitoneal Influenza A Virus Infection in Mice

**DOI:** 10.3389/fmicb.2020.01713

**Published:** 2020-07-17

**Authors:** Avishekh Gautam, Madhav Akauliya, Bikash Thapa, Byoung Kwon Park, Dongbum Kim, Jinsoo Kim, Keunwook Lee, Kyung Chan Choi, Joon-Yong Bae, Man-Seong Park, Younghee Lee, Hyung-Joo Kwon

**Affiliations:** ^1^Department of Microbiology, College of Medicine, Hallym University, Chuncheon, South Korea; ^2^Institute of Bioscience and Biotechnology, Hallym University, Chuncheon, South Korea; ^3^Center for Medical Science Research, College of Medicine, Hallym University, Chuncheon, South Korea; ^4^Department of Biomedical Science, College of Natural Sciences, Hallym University, Chuncheon, South Korea; ^5^Department of Pathology, Hallym University Sacred Heart Hospital, Chuncheon, South Korea; ^6^Department of Microbiology, College of Medicine and the Institute for Viral Diseases, Korea University, Seoul, South Korea; ^7^Department of Biochemistry, College of Natural Sciences, Chungbuk National University, Cheongju, South Korea

**Keywords:** abdominal organs, chemokines, cytokines, infection, influenza A virus, organ failure

## Abstract

In humans, respiratory infections with influenza A viruses can be lethal, but it is unclear whether non-respiratory influenza A infections can be equally lethal. Intraperitoneal infection makes the abdominal and pelvic organs accessible to pathogens because of the circulation of peritoneal fluid throughout the pelvis and abdomen. We found that high-dose intraperitoneal infection in mice with influenza A viruses resulted in severe sclerosis and structural damage in the pancreas, disruption of ovarian follicles, and massive infiltration of immune cells in the uterus. The intraperitoneal infections also caused robust upregulation of proinflammatory mediators including IL-6, BLC, and MIG. In addition, low-dose intraperitoneal infection with one influenza strain provided cross-protection against subsequent intraperitoneal or intranasal challenge with another influenza strain. Our results suggest that low-dose, non-respiratory administration might provide a route for influenza vaccination. Furthermore, these results provide insight on the pathological role of influenza A viruses in high-risk patients, including women and diabetic individuals.

## Introduction

Respiratory influenza infections account for about 290,000–650,000 deaths worldwide each year^1^. Accordingly, studies of the pathogenesis of influenza A viruses predominantly focus on the respiratory tract and related organs (Kuiken and Taubenberger, 2008; Taubenberger and Morens, 2008; Guarner and Falcon-Escobedo, 2009). Influenza infections of the respiratory tract can lead to involvement of other organs, however, such as encephalitis and pericarditis (Edelen et al., 1974; Delorme and Middleton, 1979; Protheroe and Mellor, 1991; McCullers et al., 1999; Tamaki et al., 2009). During the recent influenza H1N1 outbreak, some patients developed severe pancreatitis and multi-organ dysfunction (Habib et al., 2016). In mice that were inoculated intranasally with influenza H5N1, the virus was detected in the lungs, heart, blood, kidney, brain, spleen, and liver (Nishimura et al., 2000). In addition, highly pathogenic avian influenza viruses were reported to replicate in multiple organs of birds including the respiratory and gastrointestinal tracks (Kwon et al., 2017; Prokopyeva et al., 2019).

Epidemiological data suggest that influenza outbreaks inflict higher mortality and morbidity rates on women, especially women of reproductive age, than on men (Klein et al., 2012). Furthermore, compared with non-pregnant women, pregnant women have higher rates of hospitalization, admission to intensive care units, severe complications, and death due to influenza infection (Louie et al., 2010; Siston et al., 2010). In the 1918 influenza outbreak, 27% of 1350 pregnant women who were hospitalized due to influenza H1N1 infection died (Harris, 1919). In the 2009 influenza H1N1 outbreak in the United States, pregnant women were four-times more likely to be hospitalized because of influenza infection than the general population (Jamieson et al., 2009). Altered immune responses are proposed to be the reason for the higher morbidity and mortality of influenza infection in pregnant women (Klein et al., 2012; Rasmussen et al., 2012). Diabetic patients have also been reported to have an increased risk of morbidity and mortality due to influenza A infection (Ruiz et al., 2018). Conversely, influenza A infection is believed to increase the risk of type 1 diabetes in genetically susceptible individuals by triggering autoimmunity (Hyoty, 2016; Op de Beeck and Eizirik, 2016).

Respiratory influenza A infections can be lethal in humans. It is unclear whether exposure to influenza viruses through other routes, such as the intramuscular, intradermal, and oral routes, can be equally lethal. Intraperitoneal infection can make the abdominal and pelvic organs accessible to the pathogens because of the circulation of peritoneal fluid throughout the pelvis and abdomen (Pannu and Oliphant, 2015). In our previous study, high-dose [1 × 10^8^ plaque forming units (pfu)] intraperitoneal infection of mice with influenza A/Hongkong/4801/2014 virus (H3N2) resulted in 50% mortality, whereas low-dose (5 × 10^6^ pfu) intraperitoneal infection with influenza A A/WSN/1933 virus (WSN) induced production of IgG that was cross-reactive to other influenza A viruses, a substantial but transient depletion of B cells and macrophages, and massive neutrophil infiltration of the peritoneal cavity (Gautam et al., 2019).

We infected BALB/c mice intraperitoneally with high doses (1 × 10^8^ pfu) of WSN and H3N2 and examined the survival of the mice and the pathology and viral titers in the abdominal and pelvic organs and peritoneal fluids. We found that the mice developed severe virus infection in the abdominal and pelvic organs, leading to organ failure and greatly increased expression of chemokines and cytokines in the pancreas, ovary, and uterus. We also determined that low-dose intraperitoneal infection was cross-protective against intraperitoneal or intranasal challenge with a different influenza strain, suggesting low-dose, non-respiratory administration might be feasible as an alternative influenza vaccination. All of the experiments used female mice to focus on the cause of the increased risks associated with influenza A virus infection in female patients. The results presented here provide information about the pathology of influenza A infection in diabetic patients and women.

## Materials and Methods

### Cell Line and Viruses

Two influenza A viruses, non-mouse-adapted H3N2 and mouse-adapted WSN, were used for intraperitoneal and intranasal infection of BALB/c mice. For production of virus stocks, single-passage viruses were inoculated into the allantoic cavity of 9-day-old specific-pathogen-free (SPF) embryonated chicken eggs. After incubation at 37°C for 48 h, the allantoic cavity fluid was harvested, aliquoted, and stored at −80°C prior to use. The virus was amplified by infecting Madin Darby Canine Kidney (MDCK) cells (American Type Culture Collection, Manassas, VA, United States). MDCK cells (4 × 10^6^/dish) were cultivated in 10 cm dishes using Minimum Essential Medium (MEM) supplemented with 10% fetal bovine serum (FBS), penicillin (100 U/ml), and streptomycin (100 μg/ml) at 37°C in 5% CO_2_ atmosphere. After overnight culture, the cells were washed with PBS and influenza A virus at MOI 0.01 in MEM media containing 1 μg/ml L-tosylamide-2-phenylethyl chloromethyl ketone (TPCK)-treated trypsin was infected into each dish and then incubated at 37°C. After 1 h incubation, the supernatants were removed and then cultured for 72 h in MEM media containing 0.3% BSA. The virus culture supernatants were collected and centrifuged at 2,000 rpm for 10 min at 4°C to remove the cell debris. The quantitation of amplified viruses was performed by plaque assay. The virus preparation and cell culture procedures were performed in biosafety level 2 (BSL-2) conditions.

### Virus Plaque Assay

MDCK cells (7 × 10^5^/well) were cultured in 6-well plates overnight. The cells were washed with PBS and infected with the influenza A virus culture supernatants after ten-fold serial dilution. After 1 h incubation at room temperature with shaking applied at 15–20 min intervals, the supernatant was removed by suction. The plates were then overlaid with pre-melted 2 × DMEM/F-12 agar media [2 mM glutamine, 4% BSA, 10 mM HEPES, 2.5% sodium bicarbonate, 50 mg/ml DEAE dextran, 1 μg/ml TPCK-treated trypsin, 100 U/ml penicillin, 100 μg/ml streptomycin, and 0.6% immunodiffusion-grade agar] and incubated at 37°C for 72 h in 5% CO_2_ atmosphere. Finally, the plates were stained with 0.1% crystal violet and washed after 1 h, and virus titers were determined based on the number of plaques formed (Rhee et al., 2012).

### Ethics Statement

Animal experiments were performed in accordance with the recommendations of the Guide for the Care and Use of Laboratory Animals of the National Veterinary Research & Quarantine Service of Korea. All animal experiments were approved by the Institutional Animal Care and Use Committee of Hallym University (Permit Number: Hallym 2017-41, 2018-66, and 2019-25). To minimize pain and suffering, the mice were anesthetized by 1–2% isoflurane inhalation (JW Pharmaceutical, Seoul, South Korea). The mice were euthanized by CO_2_ inhalation if they lost 25% of their baseline adult body weight or if they showed evidence of debilitation, pain or distress, hunched posture, rough hair coat, reduced food consumption, emaciation, inactivity, ambulation difficulty, or respiratory problems. The mice were sacrificed by CO_2_ inhalation after termination of the experiments.

### Animals, Infection, and Challenge

Eight-week-old BALB/c (H-2^b^) mice (Nara Biotech, Inc., Seoul, South Korea) were used for this study. Mice (*n* = 5/each group) were infected intraperitoneally or intranasally with H3N2 or WSN at the indicated doses (1 × 10^5^ to 1 × 10^8^ pfu per mouse). For challenge experiments, mice (*n* = 10/each group) were infected intraperitoneally first with 5 × 10^6^ pfu of either H3N2 or WSN and then challenged 7 days later with 1 × 10^8^ pfu of whichever virus they were not infected with initially. The mice were maintained under SPF conditions in a controlled environment (20–25°C, 40–45% humidity, 12-h light/dark cycle; *ad libitum* access to food and water). All experimental procedures were carried out under stringent animal biosafety level 2 (ABL-2) conditions in the Hallym Clinical and Translational Science Institute in accordance with the recommendation of Institutional Biosafety Committee of Hallym University.

### Determination of Virus Titers in Tissues

Liver, lung, kidney, pancreas, spleen, ovary, and uterus were harvested 24 h or 4 days after intraperitoneal infection. Each tissue was collected in a 2 ml Eppendorf tube (Eppendorf, Hamburg, Germany) containing 1 ml PBS and stainless-steel beads (Qiagen, Hilden, Germany) and homogenized using Tissue Lyser II (Qiagen). After centrifugation for 5 min at 13,000 rpm, the supernatants were separated and serially diluted by tenfold in PBS. The diluted supernatants were then transferred to six-well plates containing MDCK cell monolayers. The quantitation of the viruses was performed by plaque assay.

### ELISA

Eight-week-old BALB/c mice were intraperitoneally infected with 1 × 10^6^, 1 × 10^7^, 5 × 10^7^, or 1 × 10^8^ pfu H3N2. After 7 days, peritoneal cavity fluids and sera were obtained. Ninety-six-well immunoplates (Nunc^TM^, Roskilde, Denmark) were coated with H3N2 (1 × 10^5^ pfu/well) in carbonate buffer (pH 9.6) and incubated overnight at 4°C. The coated plates were then washed three times with PBST (0.1% tween 20 in PBS). Appropriately diluted peritoneal cavity fluids and sera were then transferred to the wells and incubated for 2 h at room temperature. After incubation, the plates were washed and treated with either horseradish peroxidase (HRP)-conjugated goat anti-mouse IgG or goat anti-mouse IgM. IgG subclasses IgG1, IgG2a, IgG2b and IgG3 were detected with respective HRP-conjugated goat anti-mouse IgG1/IgG2a/IgG2b/IgG3 antibodies. All HRP-conjugated anti-mouse IgG or IgM antibodies were purchased from Southern Biotechnology Associates, Inc. (Birmingham, AL, United States). After incubation for 1 h at room temperature, the plates were washed five times with PBST, and the colorimetric reaction was developed using the substrate 3,3′,5,5′-tetramethylbenzidine (TMB; Kirkegaard and Perry Laboratories, Gaithersburg, MD, United States). After optimal color development, the reaction was stopped using a stop solution (Sera Care Life Sciences, Inc. Milford, MA, United States), and the absorbance was measured at 450 nm with a Spectra Max 250 microplate reader (Molecular Devices, Sunnyvale, CA, United States).

### Hematoxylin and Eosin Staining

Pancreas, spleen, uterus, and ovary were excised from mice. The tissues were fixed with 4% paraformaldehyde, subjected to alcohol dehydration, and embedded in paraffin as described previously (Rhee et al., 2012). The paraffin-embedded tissues were sectioned into 5 μm slices using a microtome (Leica RM2235, Nussloch, Germany). The tissue sections were mounted on glass slides and allowed to dry overnight at 40°C. The slides were further incubated for 30 min at 60°C to melt the paraffin. The slides were then further deparaffinized with xylene for 30 min and subjected to rehydration steps involving treatment with decreasing ethanol concentrations from 100 to 70% in distilled water. After rehydration, the slides were stained with Gill’s Hematoxylin V (Muto Pure Chemicals, Tokyo, Japan), washed under flowing tap water for 15 min, and counterstained with Eosin Y solution (Sigma-Aldrich, St. Louis, MO, United States). The slides were then dehydrated with a series of increasing concentrations of ethanol (70–100%). Then, the slides were treated with xylene and mounted with a 6:4 solution of Malinol (Muto Pure Chemicals) and xylene. Finally, images of the stained sections were captured using an Eclipse E200 microscope (Nikon, Tokyo, Japan).

### Immunohistochemistry

Formalin-fixed, paraffin-embedded tissue sections were prepared, deparaffinized, and rehydrated. The rehydrated slides were incubated with 3% H_2_O_2_ (Thermo Scientific, Waltham, MA, United States. Catalogue No: TA-125-H2O2Q) for 15 min. Then, antigen retrieval was carried out by boiling the slides in citrate buffer (pH 6.0: ScyTek Laboratories, West Logan, UT, United States) for 15 min. The slides were then washed three times with PBST and subjected to sequential blocking with avidin blocker, biotin blocker (Vector Laboratories, Burlingame, CA, United States. Catalog No: SP-2001), and normal horse serum (Vector Laboratories, Catalog No: S-2000), each for 15 min. Then, each slide was incubated with rabbit anti-cleaved caspase-3 antibody (Cell Signaling Technology, Danvers, MA, United States, Catalog No: 9661S) for 2 h. The slides were then washed and incubated with biotinylated horse anti-rabbit antibody (Vector Laboratories, Catalog No: BP 1100) for 30 min. Following incubation, the slides were washed and treated with HRP-streptavidin (Vector Laboratories, Catalog No: PK-6100). After a 30 min incubation, the enzyme-substrate reaction was developed using 3, 3′-diaminobenzidine (DAB, Thermo Fisher Scientific). The slides were further counterstained with Gill’s Hematoxylin V, dehydrated (70–100% ethanol treatment), treated with xylene, and mounted. Finally, images were taken with an Eclipse E200 microscope. All staining procedures were carried out at room temperature.

### Measurement of Cytokines and Chemokines

Pancreas, spleen, ovaries, uterus, blood, and peritoneal cavity fluids were harvested 24 h or 4 days after intraperitoneal infection with 1 × 10^8^ pfu WSN. Each tissue was homogenized using Tissue Lyser II (Qiagen). The supernatants were collected after centrifugation for 5 min at 13,000 rpm. The samples were then serially diluted in assay diluent buffer, and the amounts of cytokines and chemokines were measured using the Cytometric Bead Array (CBA) Mouse Th1/Th2/Th17 Cytokine Kit (BD Biosciences, San Jose, CA, United States. Catalog No: 560485) and the LEGENDplex^TM^ Mouse Proinflammatory Chemokine Panel (BioLegend, San Diego, CA, United States. Catalog No: 740451) in accordance with the manufacturers’ instructions. The CBA Mouse Th1/Th2/Th17 Cytokine Kit contained capture beads to measure the levels of IL-2, IL-4, IL-6, IL-10, IL-17A, TNF, and IFN-γ. The Mouse Proinflammatory Chemokine Panel consists of two sets of beads: Beads A classification, RANTES (CCL5), MIP3-alpha (CCL20), Eotaxin (CCL11), TARC (CCL17), KC (CXCL1), and MCP-1 (CCL2); Beads B classification, MIG (CXCL9), IP-10 (CXCL10), MIP-1alpha (CCL3), MIP-1beta (CCL4), BLC (CXCL13), LIX (CXCL5), and MDC (CCL22). After incubation with the capture beads and detection reagents, the samples were analyzed by FACSCalibur (BD Bioscience) and quantified using the LEGENDplex^TM^ software, version 7.0 (BioLegend).

### Statistical Analysis

Results are shown as the mean ± standard deviation. The statistical significance of differences between two samples was evaluated using Student’s *t*-test with *P* < 0.05 as the threshold for statistical significance.

## Results

### Immune Responses to High-Dose Intraperitoneal Influenza A Infection

**FIGURE 1:**
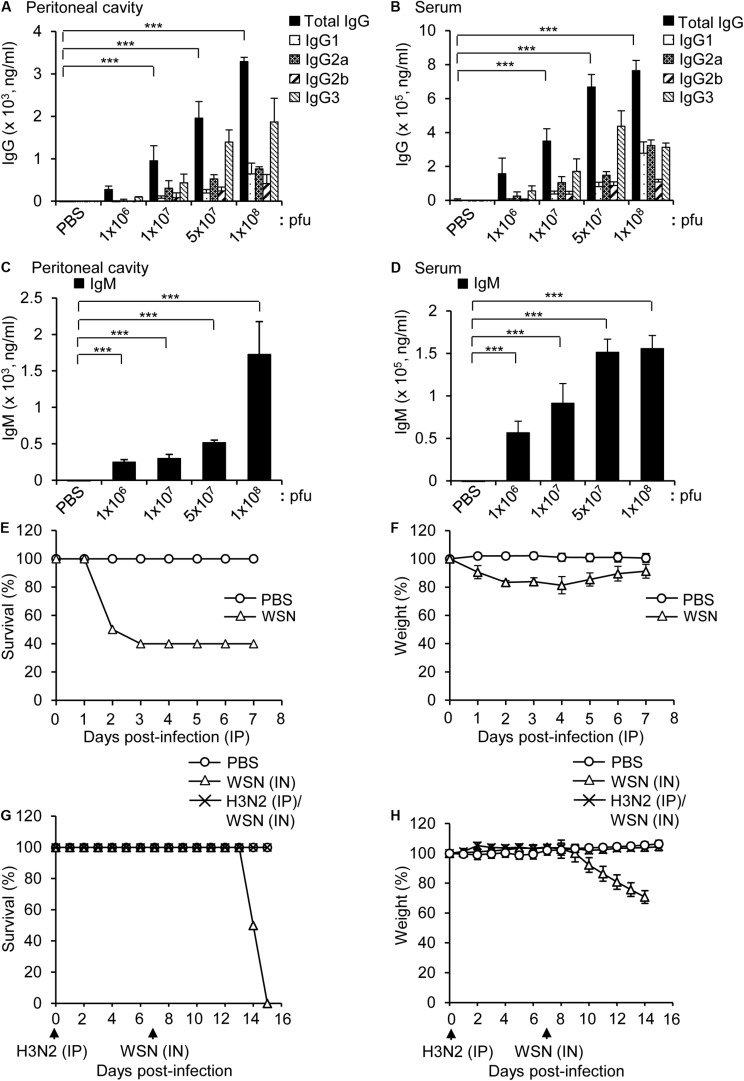
Antibody production in mice infected intraperitoneally with influenza A/Hongkong/4801/2014 virus and cross-protection against intranasal challenge with influenza A/WSN/1933 virus. **(A–D)** BALB/c mice were infected intraperitoneally with PBS (control) or different doses of A/Hongkong/4801/2014 virus (H3N2; *n* = 5/group except *n* = 10 for 1 × 10^8^ pfu). Peritoneal cavity supernatants and sera were harvested after 7 days. **(A,B)** Quantities of virus-specific IgG subclasses in the peritoneal cavity **(A)** and sera **(B)** determined by ELISA. **(C,D)** Quantities of virus-specific IgM in the peritoneal cavity **(C)** and sera **(D)** determined by ELISA. **(E,F)** BALB/c mice (*n* = 10) were intraperitoneally infected with PBS or 1 × 10^8^ pfu influenza A/WSN/1933 virus (WSN) and monitored for 7 days. Percentage survival **(E)** and body weight **(F)** for 7 days were measured. **(G,H)** BALB/c mice (*n* = 10) were infected intraperitoneally (IP) with 5 × 10^6^ pfu H3N2. After 7 days, the mice were challenged intranasally (IN) with 1 × 10^5^ pfu WSN. The survival **(G)** and body weight **(H)** of the mice were recorded for the next 8 days. **P < 0.005, ***P < 0.0005.

BALB/c mice were intraperitoneally infected with different doses of H3N2 and the virus-reactive antibody production (IgG and IgM) was measured by ELISA 7 days post infection. The levels of influenza H3N2-reactive IgG and IgM increased in the peritoneal cavity fluids and sera in a dose-dependent manner (Figures 1A–D), although the number of B cells in the peritoneal cavity and bone marrow decreased after infection with the highest dose (1 × 10^8^ pfu) of the virus (Gautam et al., 2019). The amount of IgG3 was higher than those of other IgG classes, which is similar to the IgG repertoire after intraperitoneal infection with WSN (Gautam et al., 2019).

Intraperitoneal infection of BALB/c mice with a high dose (1 × 10^8^ pfu) of WSN induced 40% mortality (Figures 1E,F). In a previous study, intraperitoneal infection of BALB/c mice with a low dose (5 × 10^6^ pfu) of WSN had a protective effect against subsequent intraperitoneal infection with a high dose (1 × 10^8^ pfu) of H3N2, a dose that was fatal in previously uninfected mice (Gautam et al., 2019). We performed a similar experiment in which we first infected mice intraperitoneally with 5 × 10^6^ pfu H3N2 and then infected them intranasally with a potentially lethal dose (1 × 10^5^ pfu) WSN. The results showed that the low-dose intraperitoneal infection with H3N2 had a protective effect against subsequent lethal-dose intranasal infection with WSN (Figures 1G,H).

### High-Dose Intraperitoneal Influenza A Infection Caused Sclerosis of the Pancreas and Abdomen

**FIGURE 2:**
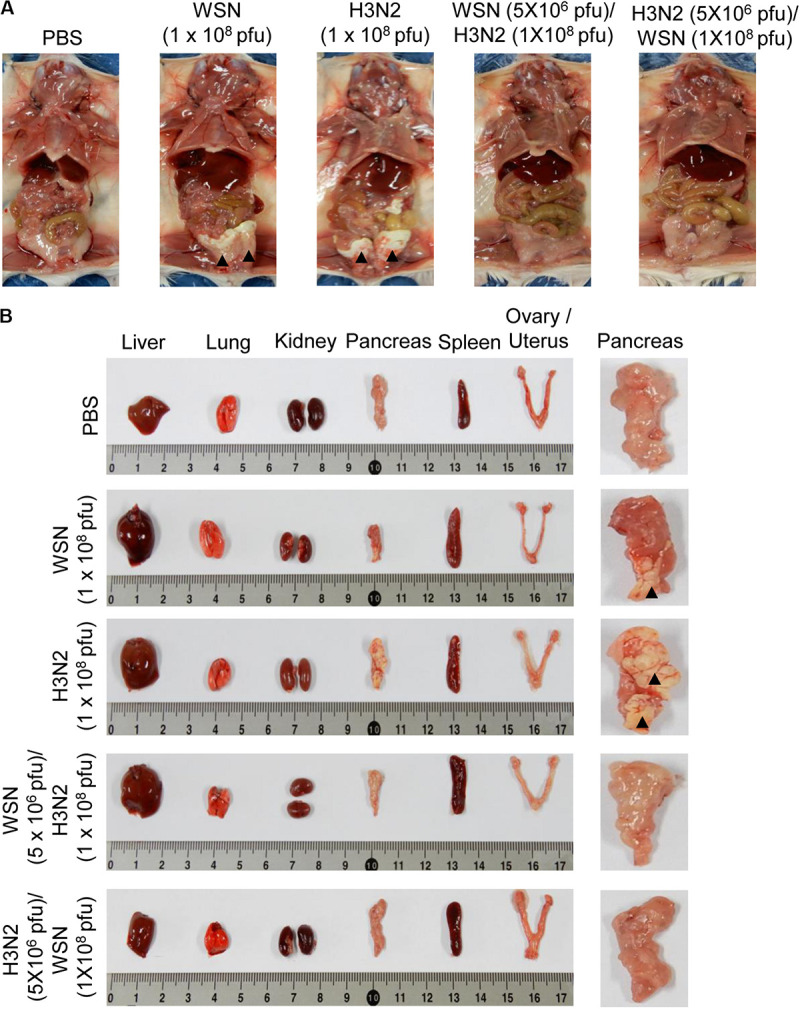
Physical condition of the peritoneal cavity and organs after high-dose intraperitoneal infection with influenza A/Hong Kong/4801/2014 virus and/or influenza A/WSN/1933 virus infection. BALB/c mice were intraperitoneally infected with PBS or 1 × 10^8^ pfu influenza A/WSN/1933 virus (WSN; *n* = 10/group) or influenza A/Hongkong/4801/2014 virus (H3N2; *n* = 10/group). After 4 days, the surviving mice were sacrificed. Other BALB/c mice were intraperitoneally infected with 5 × 10^6^ pfu WSN or H3N2 (*n* = 5/group). After 7 days, the mice were challenged intraperitoneally with 1 × 10^8^ pfu of the virus that they were not infected with previously. The mice were sacrificed 4 days later. **(A)** Images of the exposed peritoneal cavity. Arrowheads indicate sclerosis of the fatty tissue. **(B)** Organs collected from PBS-treated or virus-infected mice. Right panel shows magnified images of the pancreas. Arrowheads indicate sclerotic lesions.

Mice were infected intraperitoneally with 1 × 10^8^ pfu WSN or H3N2 and sacrificed 4 days later to determine the effects of the infection on the abdominal and pelvic organs and tissues. In addition, to investigate the cross-protective effects of influenza A infection, we infected mice intraperitoneally with 5 × 10^6^ pfu WSN or H3N2 and then challenged them intraperitoneally 7 days later with 1 × 10^8^ pfu of the virus that they were not exposed to in the initial infection. Four days after challenge, mice were sacrificed, and their abdominal tissues and organs were examined. The mice that were infected with 1 × 10^8^ pfu of either virus without prior infection displayed sclerosis of the fatty tissue covering the abdomen (Figure 2A). All of the infected mice had enlarged spleens; however, only the mice that were infected with 1 × 10^8^ pfu of either virus without prior infection had sclerotic lesions on the pancreas. By contrast, the challenged mice after initial infection, all of which survived until they were sacrificed (Figure 1G), had no physical abnormalities 4 days after challenge (Figures 2A,B). When we infected mice intraperitoneally with 5 × 10^6^ pfu influenza A virus and examined the effects of the infection on the abdominal and pelvic organs and tissues as a control, there was no physical abnormalities observed at 7 days after infection (Supplementary Figures S1A,B).

### High-Dose Intraperitoneal Influenza A Infection Caused Histological Changes in the Pancreas and Uterus

**FIGURE 3:**
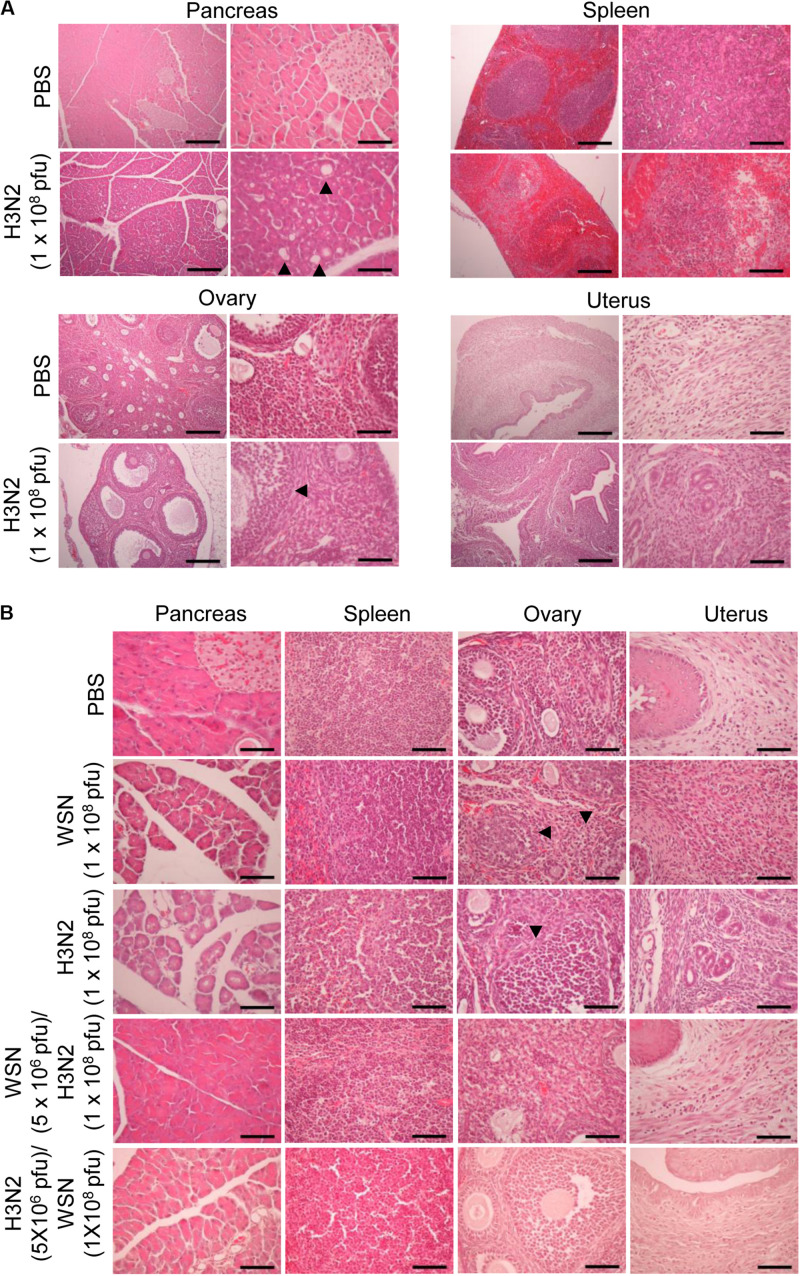
Histopathology of organ tissues after high-dose intraperitoneal infection with influenza A/Hong Kong/4801/2014 virus and/or influenza A/WSN/1933 virus. **(A)** BALB/c mice (*n* = 5/group) were intraperitoneally infected with 1 × 10^8^ pfu influenza A/Hongkong/4801/2014 virus (H3N2). After 24 h, the mice were sacrificed, and the abdominal organs were excised. H&E staining was performed on formalin-fixed, paraffin-embedded tissue sections of the pancreas, spleen, ovaries, and uterus. Arrowheads indicate fat globes in the pancreas and disruption of ovarian follicles in the ovaries. Scale bars: left panel 100 μm, right panel 25 μm. **(B)** BALB/c mice (*n* = 10/group) were intraperitoneally infected with 1 × 10^8^ pfu influenza A/WSN/1933 virus (WSN) or H3N2. After 4 days, the surviving mice were sacrificed. Other groups of BALB/c mice (*n* = 5/group) were intraperitoneally infected with 5 × 10^6^ pfu WSN or H3N2. Seven days later, the mice were challenged with 1 × 10^8^ pfu of the virus that they were not infected with previously. The mice were sacrificed 4 days later. Formalin-fixed, paraffin-embedded 5-μm tissue sections of the pancreas, spleen, ovaries, and uterus were subjected to H&E staining. Arrowheads indicate disruption of ovarian follicles in the ovaries. Scale bars, 25 μm.

To determine the histological state of different organs and evaluate possible cause of death induced by high dose viral infection, mice were sacrificed, and different organs were excised 24 h or 4 days after infection. H&E staining of formalin-fixed, paraffin-embedded pancreas sections taken 24 h after initial infection with influenza H3N2 revealed severe disruption of the acinar glands and islets of Langerhans and the appearance of fat globes (droplets) (Figure 3A). The white pulp of the spleen exhibited erythrocyte leakage caused by disruption of the blood vessels. Compared with those of control mice, the ovaries of the virus-infected mice displayed minimal alterations; however, the uteruses of the virus-infected mice were massively infiltrated with inflammatory cells, in contrast to those of the control mice (Figure 3A). The mice that were sacrificed 4 days after initial infection with either virus displayed massive infiltration of inflammatory cells in the uterus, disruption of ovarian follicles, and severe disruption of the acinar glands and islets of Langerhans throughout the pancreatic tissue sections (Figure 3B). The spleens of those mice showed substantial recovery, however, compared with the spleens of the mice that were sacrificed 24 h after initial infection (Figure 3B). In the mice that were challenged after the initial infection, the pancreas showed no prominent abnormalities. Furthermore, the challenged mice showed reduced inflammatory-cell infiltration in the uterus compared with mice that were subjected to a single high-dose infection (Figure 3B). When we infected mice intraperitoneally with 5 × 10^6^ pfu influenza A virus as a control, there was no significant changes in the histological state of the different organs in the mice at 7 days after infection (Supplementary Figure S1C).

### Detection of Influenza A Viruses in the Abdominal and Pelvic Organs and Peritoneal Cavity After High-Dose Intraperitoneal Influenza A Infection

**FIGURE 4:**
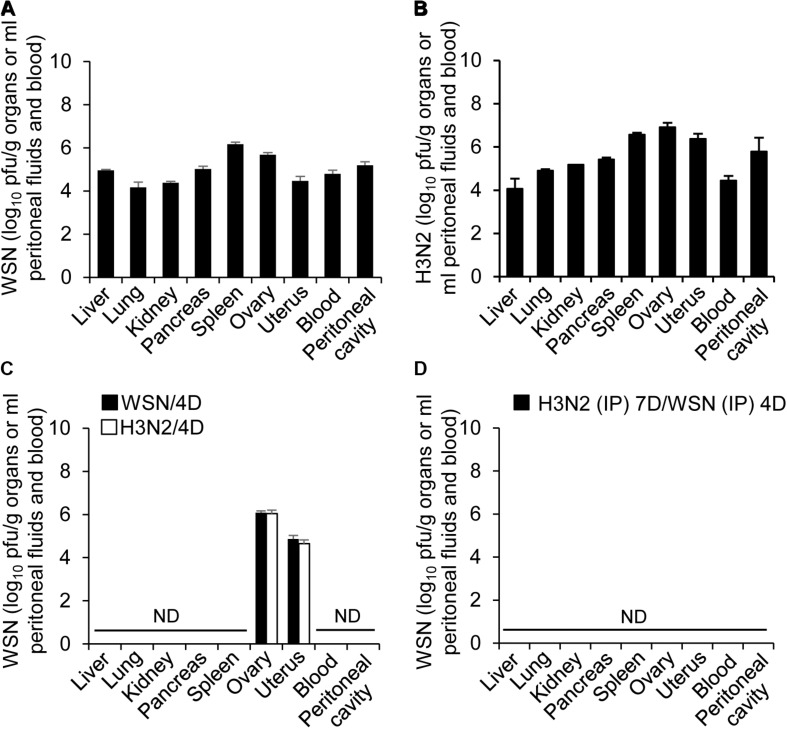
Titers of influenza A/Hong Kong/4801/2014 virus and influenza A/WSN/1933 viruses in peritoneal fluids and organs after high-dose intraperitoneal infection. **(A,C)** BALB/c mice (*n* = 15/group) were intraperitoneally infected with 1 × 10^8^ pfu influenza A/WSN/1933 virus (WSN) or influenza A/Hongkong/4801/2014 virus (H3N2). After 24 h, five mice in each group were sacrificed, and the titers of WSN **(A)** and H3N2 **(B)** in the abdominal organs and peritoneal fluids were determined by plaque assay. Four days after the initial infection, five more mice in each group (WSN/4D and H3N2/4D, respectively) were sacrificed, and the viral titers in the abdominal organs and peritoneal fluids were determined by plaque assay **(C)**. **(D)** BALB/c mice were infected intraperitoneally with 5 × 10^6^ pfu H3N2. Seven days later, the same mice were challenged with 1 × 10^8^ pfu WSN. Four days after challenge, the mice were sacrificed, and the viral titers in the abdominal organs and peritoneal fluids were determined by plaque assay. ND, no virus detected when the samples corresponding to 100% of the abdominal organs and peritoneal fluids were analyzed.

To further determine if influenza A virus, infected intraperitoneally, can affect the normal function of organs closely associated with the peritoneal cavity, it is required to examine whether influenza A virus can infect the organs. Therefore, blood, peritoneal cavity fluids, liver, kidney, pancreas, spleen, ovary and uterus were harvested from the mice at 24 h or 4 days after infection with WSN or H3N2 or after initial infection with one of those viruses and subsequent challenge with the other virus. The organs were lysed, and virus titers were measured by plaque assay. All of the organs and fluids harvested 24 h after initial infection yielded viral titers (Figures 4A,B). In the organs and fluids harvested 4 days after initial infection, there were high viral titers in the ovaries, low titers in the uterus, and no detectable virus titers in the other organs and fluids (Figure 4C). To test the possibility that the organs contained very low numbers of virus particles that could potentially replicate in the future, we isolated cells from the organs of uninfected mice and those of mice infected with 5 × 10^6^ pfu of either virus and then allowed any viruses present in the cells to replicate in a plaque assay. We did not detect any viral amplification in the cells isolated from any of the organs (data not shown). When the mice were infected with 5 × 10^6^ pfu H3N2 prior to challenge with 1 × 10^8^ pfu WSN, there were no viral titers in the examined organs 4 days after challenge (Figure 4D). In a previous study, we infected mice intraperitoneally with low-dose of virus (5 × 10^6^ pfu) and could not detect the virus in the spleen, heart, liver, lung, kidney, and peritoneal cavity from the mice when we examined at 5–14 days post-infection (Gautam et al., 2019). We also could not detect the virus in pancreas, uterus, and ovary of the mice at 7 days after infection (Supplementary Figure S1D).

### High-Dose Intraperitoneal Influenza A Infection Led to High Blood Glucose Levels and Apoptosis of Pancreatic Cells

**FIGURE 5:**
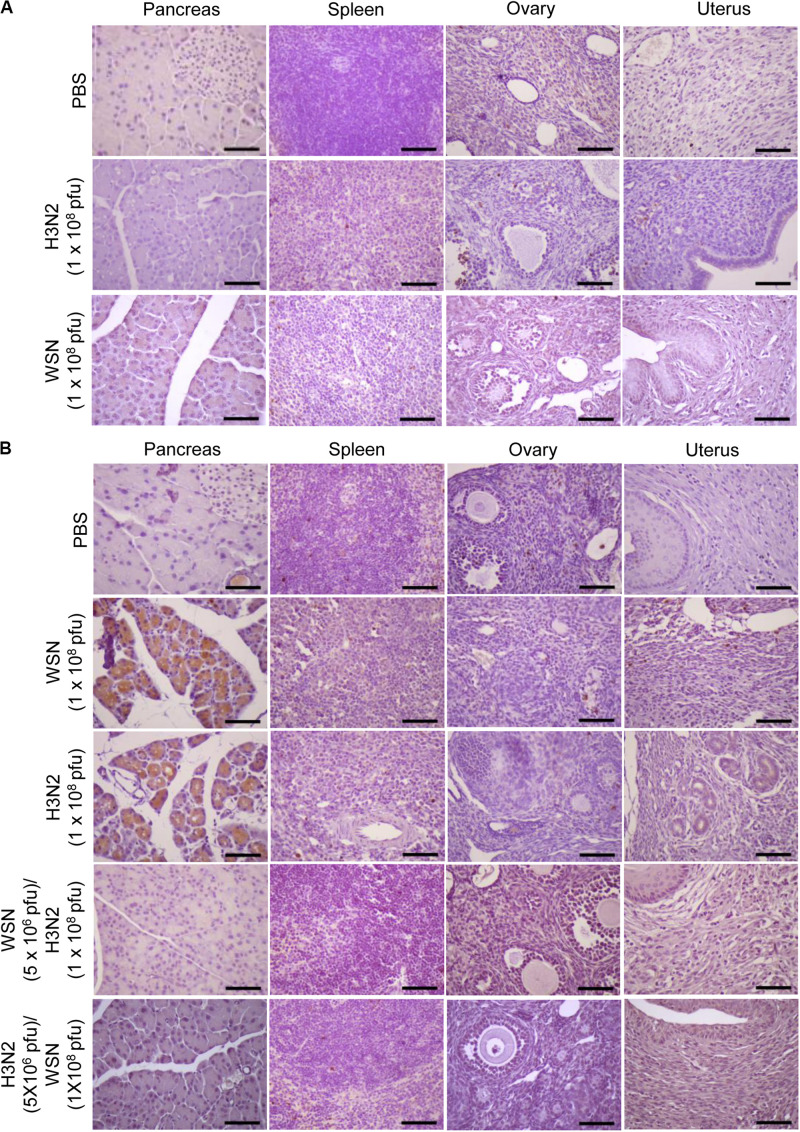
Apoptosis in abdominal organs following intraperitoneal infection with A/Hong Kong/4801/2014 virus and/or A/WSN/1933 virus. **(A)** BALB/c mice (*n* = 5/group) were intraperitoneally infected with 1 × 10^8^ pfu influenza A/WSN/1933 virus (WSN) or influenza A/Hongkong/4801/2014 virus (H3N2). After 24 h, the mice were sacrificed. Formalin-fixed, paraffin-embedded tissue samples of the pancreas, spleen, ovaries, and uterus were sliced to 5 μm thickness, and immunohistochemical staining was performed to detect cleaved caspase-3. **(B)** BALB/c mice (*n* = 10/group) were intraperitoneally infected with PBS or 1 × 10^8^ pfu WSN or H3N2. After 4 days, the mice were sacrificed. Another group of BALB/c mice (*n* = 5/group) were infected intraperitoneally with 5 × 10^6^ pfu WSN or H3N2. Seven days later, the same mice were challenged with 1 × 10^8^ pfu of the virus that they were not infected with previously. The mice were sacrificed after 4 days. Formalin-fixed, paraffin-embedded tissue samples of the pancreas, spleen, ovaries, and uterus were sliced to 5 μm thickness, and immunohistochemical staining was performed to detect cleaved caspase-3. Scale bars, 25 μm.

Immunohistochemistry of paraffin-embedded tissue sections was performed using anti-cleaved caspase-3 antibody to determine if intraperitoneal WSN or H3N2 infection induced apoptosis after 24 h (Figure 5A) or 4 days (Figure 5B). Mice infected with either virus were positive for cleaved caspase-3 in the pancreas 4 days after infection (Figure 5B) but not 24 h after infection (Figure 5A); however, they showed no signs of apoptosis in the ovaries or uterus. Mice that were infected intraperitoneally with a low dose of one virus and then challenged intraperitoneally with a high dose of the other virus showed no evidence of apoptosis in the pancreas or spleen 4 days after challenge (Figure 5B).

Because H3N2 induced massive structural damage (Figure 3B) and apoptosis (Figure 5B) in the pancreas, we asked whether it had any effect on glucose metabolism. We measured the fasting glucose levels in mice infected with influenza H3N2 (5 × 10^6^ pfu and 1 × 10^8^ pfu) at the time of initial infection and again 4 days later. We found that the blood glucose levels were markedly increased 4 days after H3N2 infection (1 × 10^8^ pfu) compared with those at the time of initial infection. In contrast, there was no change in the glucose levels 4 days after 5 × 10^6^ pfu H3N2 infection (Supplementary Figure S2).

### The Cells of the Abdominal and Pelvic Organs Possess Specific Receptors for Influenza A Virus

To determine the binding potential of influenza A viruses in the abdominal and pelvic organs, cells were isolated from the pancreas, spleen, ovary, and uterus of uninfected mice and the expression of specific receptors for influenza A virus was analyzed by flow cytometry using SNA (for α-2, 6-Gal sialic acid) and MAL II (for α-2, 3-Gal sialic acid). The cells of all four organs expressed both α-2, 6-Gal and α-2, 3-Gal sialic acid residues (Supplementary Figures S3A,B), indicating that they are susceptible to binding by avian and human influenza A viruses. To determine the *in vitro* response of the isolated cells to challenge with influenza A virus, we treated them with H3N2 for 72 h and analyzed the degree of apoptosis using Annexin V staining. We observed apoptosis in the cells of the pancreas but not in those of the spleen, ovary, or uterus (Supplementary Figure S3C).

### High-Dose Intraperitoneal Influenza A Infection Induced Cytokine and Chemokine Expression

**FIGURE 6:**
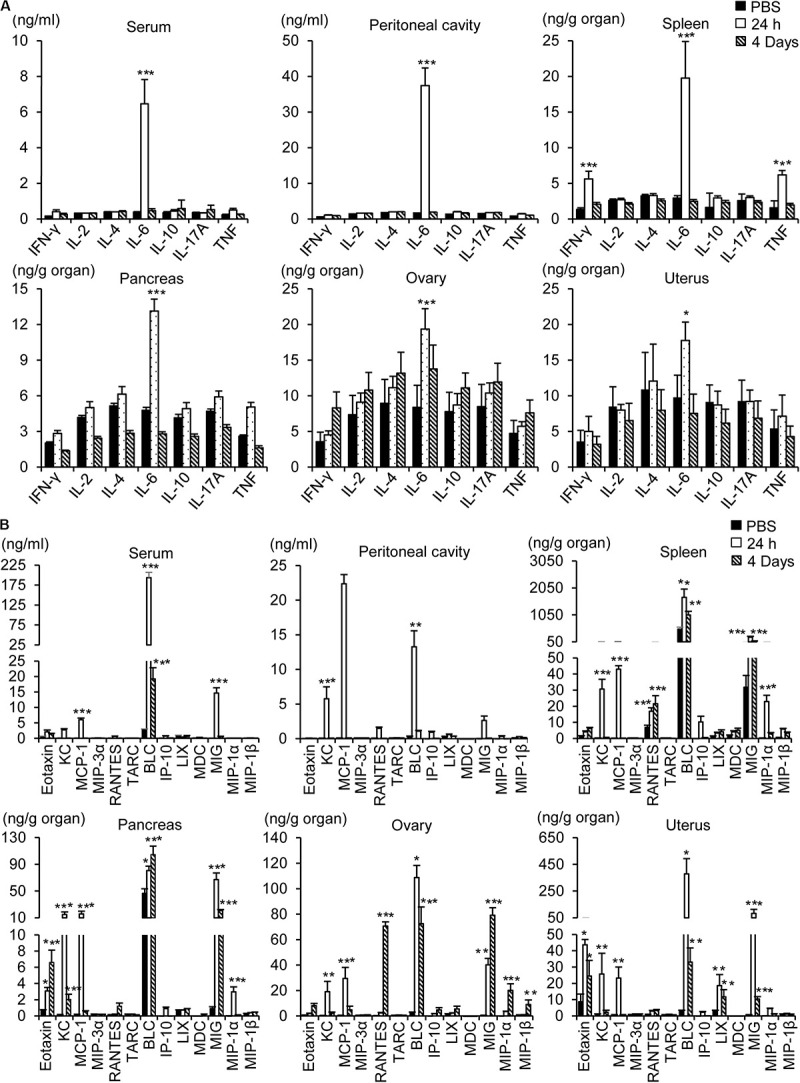
Measurement of cytokines and chemokines. Pancreas, spleen, ovary, uterus, blood, and peritoneal cavity fluids were collected from BALB/c mice 24 h or 4 days after intraperitoneal infection with influenza A/WSN/1933 virus. Cytokines **(A)** and chemokines **(B)** in organ lysates, sera, and peritoneal fluids were quantified using a CBA mouse Th1/Th2/Th17 cytokine kit (BD Biosciences) and a LEGENDplex^TM^ Mouse Proinflammatory Chemokine Panel (BioLegend). *p < 0.05, **P < 0.005, and ***P < 0.0005 indicate statistical significance compared with PBS injection.

To determine whether inflammation contributes to the pathogenesis of influenza A infection in the abdominal and pelvic organs, levels of cytokines and chemokines were measured using a multiplex cytokine array. Intraperitoneal infection with 1 × 10^8^ pfu WSN caused an increase in the level of the proinflammatory cytokine IL-6 in the peritoneum, blood, spleen, pancreas, ovaries, and uterus after 24 h (Figure 6A); however, the levels of other proinflammatory cytokines such as IFN-γ and TNF-α were not increased in the abdominal tissues after the infection.

Intraperitoneal infection with WSN caused robust upregulation of proinflammatory chemokines in the abdominal tissues, peritoneum, and blood (Figure 6B). The most prominent induction was that of B lymphocyte chemoattractant (BLC), which was sustained for 4 days post infection. Similarly, monokine induced by gamma interferon (MIG) was upregulated in the pancreas, ovary, and uterus after WSN infection and remained at a high level in those organs for 4 days after infection. On the other hand, the levels of keratinocyte chemoattractant (KC) and monocyte chemoattractant protein-1 (MCP-1) were increased 24 h post infection but not 4 days post infection (Figure 6B). The production of eotaxin was increased after WSN infection in the pancreas and ovaries but not in the blood or peritoneum. The virus-induced expression of RANTES was confined to the ovary and spleen and occurred later than that of the other chemokines (Figure 6B).

## Discussion

Pathological disturbances of the respiratory tract are regarded as the major cause of mortality and morbidity during influenza A epidemics and pandemics. However, several studies have reported that influenza A infections can involve other sites such as the bone marrow, brain, heart, and pancreas (Edelen et al., 1974; Delorme and Middleton, 1979; Protheroe and Mellor, 1991; McCullers et al., 1999; Nishimura et al., 2000; Tamaki et al., 2009; Capua et al., 2013; Habib et al., 2016). To understand the effect of influenza A infection on organs that are not considered to be primary targets of the virus, we infected mice intraperitoneally with WSN and/or H3N2. Intraperitoneal infection of mice with influenza A virus exposes the organs of both the abdomen and the pelvis to the virus.

Turkeys infected intranasally with influenza H7N1 virus and influenza H7N3 virus exhibited histopathological changes in the pancreas, including infiltration by inflammatory cells, necrosis of acinar cells, fibrosis, and ductal hyperplasia (Capua et al., 2013). We found that high-dose intraperitoneal infection of mice with H3N2 or WSN resulted in sclerosis of the adipose tissue covering the abdomen in the peritoneal cavity, sclerotic lesions of the pancreas, and massive destruction of the pancreatic structure. Furthermore, we observed apoptosis in pancreatic tissue sections 4 days post infection. It has been reported that influenza A infection induces cell death through apoptosis (Chen et al., 2001), necrosis (Mosavi et al., 2015), and necroptosis (Sridharan and Upton, 2014; Nogusa et al., 2016). Influenza A virus induces apoptosis in macrophages, dendritic cells, monocytes, and epithelial cells of the airway and lungs by intrinsic (mitochondrial-dependent) and extrinsic pathways (Atkin-Smith et al., 2018). The mitochondrial membrane proteins ANT3 and VDAC1 sense the PB1-F2 protein of the influenza H1N1 virus, triggering cytochrome c release and apoptosis induction (Chen et al., 2001; Zamarin et al., 2005). The influenza NP protein also induces apoptosis by interacting with the anti-apoptotic factor API5 and the E3 ubiquitin ligase RNF43 (Mayank et al., 2015; Nailwal et al., 2015). The influenza NS1 protein induces apoptosis via an extrinsic pathway by upregulating FasL (Schultz-Cherry et al., 2001). In order to protect the host against influenza A virus, the cytoplasmic protein RIPK3 activates the parallel pathways of FADD-driven apoptosis and MLKL-driven necroptosis (Nogusa et al., 2016). Our previous results showed that interaction of the influenza HA protein with α-2,6-linked and α-2,3-linked sialic acids in B cells and macrophages induces apoptosis of those cells in the peritoneal cavity (Gautam et al., 2019). Although we detected expression of influenza virus receptors in the pancreas, spleen, ovary, and uterus, influenza A infection only induced apoptosis in pancreatic cells. At early time points after intraperitoneal infection with H3N2 or WSN, we detected virus in the lungs, heart, blood, kidneys, pancreas, spleen, uterus, and ovaries. By contrast, 4 days after initial infection, the viruses were detectable only in the uterus and ovaries. Those results suggest that influenza A viruses that enter the host via the intraperitoneal route can circulate to various abdominal and pelvic organs but only induce apoptosis in pancreatic cells. The mice infected with H3N2 displayed hyperglycemia 4 days post infection, which was probably the result of severe structural damage to the pancreas. A previous study reported similar hyperglycemic conditions in turkeys that were infected intranasally with influenza virus (Capua et al., 2013). Our results and the previous results are in line with reports that diabetic patients are more sensitive to influenza viruses than non-diabetic patients (Ruiz et al., 2018).

High-dose intraperitoneal infection with H3N2 or WSN resulted in massive immune-cell infiltration of the uterus 4 days post infection. We also observed disruption of ovarian follicles, although there were no apoptotic signals in the ovaries of the infected mice 4 days post infection. Those results are in line with previous reports that the threat of influenza A infection is increased in women, especially during pregnancy (Harris, 1919; Jamieson et al., 2009; Louie et al., 2010; Siston et al., 2010; Klein et al., 2012; Rasmussen et al., 2012). It is hard to suppose a direct association between the ovaries and pancreas and the usual route of influenza infection, because intranasal infections do not always result in the spread of the virus to the organs of the abdominal or pelvic cavities. We suggest, however, that peritoneal infection can be used as a model system to explore the higher sensitivity of diabetic patients and women to influenza viruses.

Influenza A infection is assumed to provoke strong inflammation at the site of infection. Morbidity, pathogenesis, complications, and mortality due to influenza infection are all related to components of the innate immune system that are involved with clearance of the virus. Chemokines and their receptors are key factors in the activation and site-directed migration of leukocytes, which in turn secrete various inflammatory cytokines. Of the proinflammatory cytokines that we tested, IL-6 was the most prominently upregulated both systemically and in the abdominal tissues after intraperitoneal influenza A infection. IL-6 plays a pivotal role in the acute phase of immune responses and regulates innate and adaptive immunity to clear invading pathogens, including influenza viruses (Dienz et al., 2012; Tanaka et al., 2014). Several reports suggest that IL-6 expression is correlated with disease severity following influenza (H1N1) infection (Hagau et al., 2010; Paquette et al., 2012) and that IL-6 plays an essential role in tissue repair during viral infection (Dienz et al., 2012; Yang et al., 2017). We found that the extent of IL-6 induction after influenza A infection was much milder in the pancreas, ovary, and uterus compared with that in the spleen. That might explain the higher sensitivity of the pancreas to apoptosis and the slower viral clearance in the ovaries and uterus compared with those in other organs.

Chemokine expression and recruitment of proinflammatory leukocytes in the infected area are required for viral clearance. In the abdominal organs and blood, the level of the chemokine BLC was dramatically increased after intraperitoneal infection with WSN. BLC, also called CXCL13, is chemotactic for B cells (Ansel et al., 2002). The levels of MCP1, also called CCL2, and KC, also called CXCL1, were transiently increased in the pancreas, ovary, and uterus after influenza A infection. MCP1 exhibits chemotactic activity for monocytes and is implicated in autoimmune diseases, including psoriasis and rheumatoid arthritis, as well as infectious diseases caused by HIV and influenza virus (Takahashi et al., 2009; Narasaraju et al., 2010; Ansari et al., 2011). KC is produced at high levels during the early phase of influenza infection, resulting in neutrophil recruitment to the affected area (Tavares et al., 2017). We observed that intraperitoneal infection with WSN led to robust upregulation of MIG, also called CXCL9, which, unlike MCP1 and KC, remained at a high level in the pancreas and ovaries 4 days after infection. MIG is involved in the polarization of IFN-γ-producing type-1 T cells and the activation of inflammatory macrophages (Zohar et al., 2014), which suggests that it plays a role in training innate and adaptive immune cells to clear pathogens in the later stages of infection. Therefore, we speculate that in combination with the immediate early responses of MCP1 and KC chemotaxis, the differentiation and activation of immune cells induced by cytokines and chemokines elicit appropriate immunity to influenza virus concurrently with the occurrence of tissue damage in the abdominal organs.

Intraperitoneal infection provides a direct way to study the pathophysiological changes caused by influenza A infection at non-respiratory sites. Intraperitoneal inoculation with high doses of WSN (Gautam et al., 2019) or H3N2 resulted in high titers of anti-viral antibodies in the abdominal and pelvic organs. Furthermore, immunological responses induced by low-dose intraperitoneal infection with one strain of influenza A virus induced an effective defense against future intraperitoneal or intranasal reinfection or challenge with a high dose of another strain of influenza A virus. The protective effect of prior infection was clearly confirmed in the context of survival, viral clearance, and protection of sensitive organs such as the pancreas, ovaries, and uterus. As intraperitoneal infection of low-dose influenza A virus induced production of antibodies cross-reactive to other influenza viruses (Gautam et al., 2019), humoral immunity is evidently involved in this protective effect. Considering the large increase of CD8^+^ T cell population in the peritoneal cavity after low-dose intraperitoneal infection with WSN (Gautam et al., 2019), it is likely that cell mediated immunity also contributes to protection. It was previously reported that CD8^+^ memory T cells intraperitoneally primed with laboratory-adapted influenza viruses are involved in the protection from respiratory challenge with extremely virulent influenza viruses (Riberdy et al., 1999; Christensen et al., 2000). Involvement of heterologous cell mediated immune response was also reported in avian influenza viruses (Nfon et al., 2012). Considering that the mediastinal lymph nodes (MLN) were presumed to be the primary sites of recall response in the intranasal infection of influenza viruses (Flynn et al., 1998), there can be some differences in the efficacy of T cell responses upon intranasal challenge depending on the prior infection routes. Although the intraperitoneal route is difficult to apply in human clinics, and high-dose intraperitoneal infection caused organ injury in mice, our results suggest that a trial to find alternative route of vaccination is meaningful to pursue. Intranasal administration of a live-attenuated influenza A virus vaccine, inducing protective immunity and enhancing mucosal immunity (Bodewes et al., 2013; Maroof et al., 2014), is convenient from a practical standpoint. However, the efficacy of this strategy is still controversial (Caspard et al., 2017; Gill and Schlaudecker, 2018). More detailed comparative studies of vaccination by various routes in animals might reveal ways to develop more efficacious and reliable influenza vaccines.

## Data Availability Statement

All data needed to evaluate the conclusions in the article are present in the article/Supplementary Materials. Virus strains are available from the authors. Additional data related to this article may be requested from the authors.

## Ethics Statement

The animal study was reviewed and approved by Institutional Animal Care and Use Committee of Hallym University.

## Author Contributions

H-JK conceived of the project. H-JK, M-SP, and YL designed the experiments and wrote the manuscript. AG, MA, BT, BP, DK, JK, and J-YB carried out the experiments. H-JK, M-SP, KL, KC, and YL analyzed the data. All authors approved the final version of the manuscript.

## Conflict of Interest

The authors declare that the research was conducted in the absence of any commercial or financial relationships that could be construed as a potential conflict of interest.

## Footnotes

1 https://www.who.int/mediacentre/news/statements/2017/flu/en/
