# Supplementary material for: Abdominal and Pelvic Organ Failure Induced by Intraperitoneal Influenza A Virus Infection in Mice
Source: Front Microbiol. 2020 Jul 17;11:1713. doi: 10.3389/fmicb.2020.01713 (PMC7379156; doi:10.3389/fmicb.2020.01713)
Supplement: Supplementary file 1 [file Data_Sheet_1.pdf]

Supplementary Material

Supplementary Methods

Animals and infection

Eight-week-old BALB/c (H-2^b) mice (Nara Biotech, Inc., Seoul, Korea) were infected intraperitoneally with 5×10^6 pfu H3N2 or WSN. After 7 days, the mice were sacrificed, and the abdominal organs were excised, and then H&E staining was performed. Virus titers in pancreas, uterus, and ovary were determined by plaque assay.

Lectin staining

Cells were isolated from the pancreas, spleen, uterus, and ovaries of BALB/c mice. Erythrocytes were removed with RBC lysis buffer and then washed with FACS buffer. The cells were then stained with either biotinylated *Maackia amurensis* lectin II (MAL II; Vector Laboratories, Catalogue No: B 1265) or fluorescein-conjugated *Sambucus Nigra* agglutinin (SNA; Vector Laboratories, Catalogue No: L-1300) and incubated for 1 h at 4°C. SNA and MAL II preferentially bind to α-2, 6-Gal (human flu-specific receptor) and α-2, 3-Gal (avian flu-specific receptor) sialic acid residues, respectively. After incubation, the cells were washed with FACS buffer. The MAL II-treated samples were further incubated with APC-conjugated streptavidin (Thermo Fisher Scientific, Catalogue No: SA1005) for 30 min. After washing, lectin binding was detected by flow cytometry (BD FACSCalibur™, BD Biosciences, San Jose, CA, USA).

Annexin V staining

Isolated cells from the pancreas, spleen, ovaries, and uterus of BALB/c mice were seeded on 24-well plates (2×10^6 cells/well) and treated with 1×10^6 pfu H3N2. After incubation for 72 h, the cells were collected into 5 ml polystyrene round-bottomed tubes and washed with FACS buffer. The cells were then blocked with FcγRII/III antibody at 4°C for 20 min and incubated with 5 µl APC-conjugated Annexin V (Thermo Fisher Scientific, Catalogue No: 17-8007-74) for 15 min at room temperature. Following incubation, the cells were washed, treated with 5 µl propidium iodide (Thermo Fisher Scientific, Catalogue No:00-6990-42), and analyzed by FACSCanto™ II (Becton Dickinson, Franklin Lakes, NJ, USA).

Measurement of blood glucose levels

Prior to blood collection, the mice were starved for 12 h. Blood was collected by retro-orbital puncture using heparinized capillary tubes (Chase, Scientific glass, Inc. Rockwood). A drop of blood was placed in the sample loading spot of the rapid test strip (Accu-chek performa, Roche, NSW, Australia), and the glucose level was measured using a Standard Mentor NFC (SD biosensor, Beijing, China).

Supplementary Figures

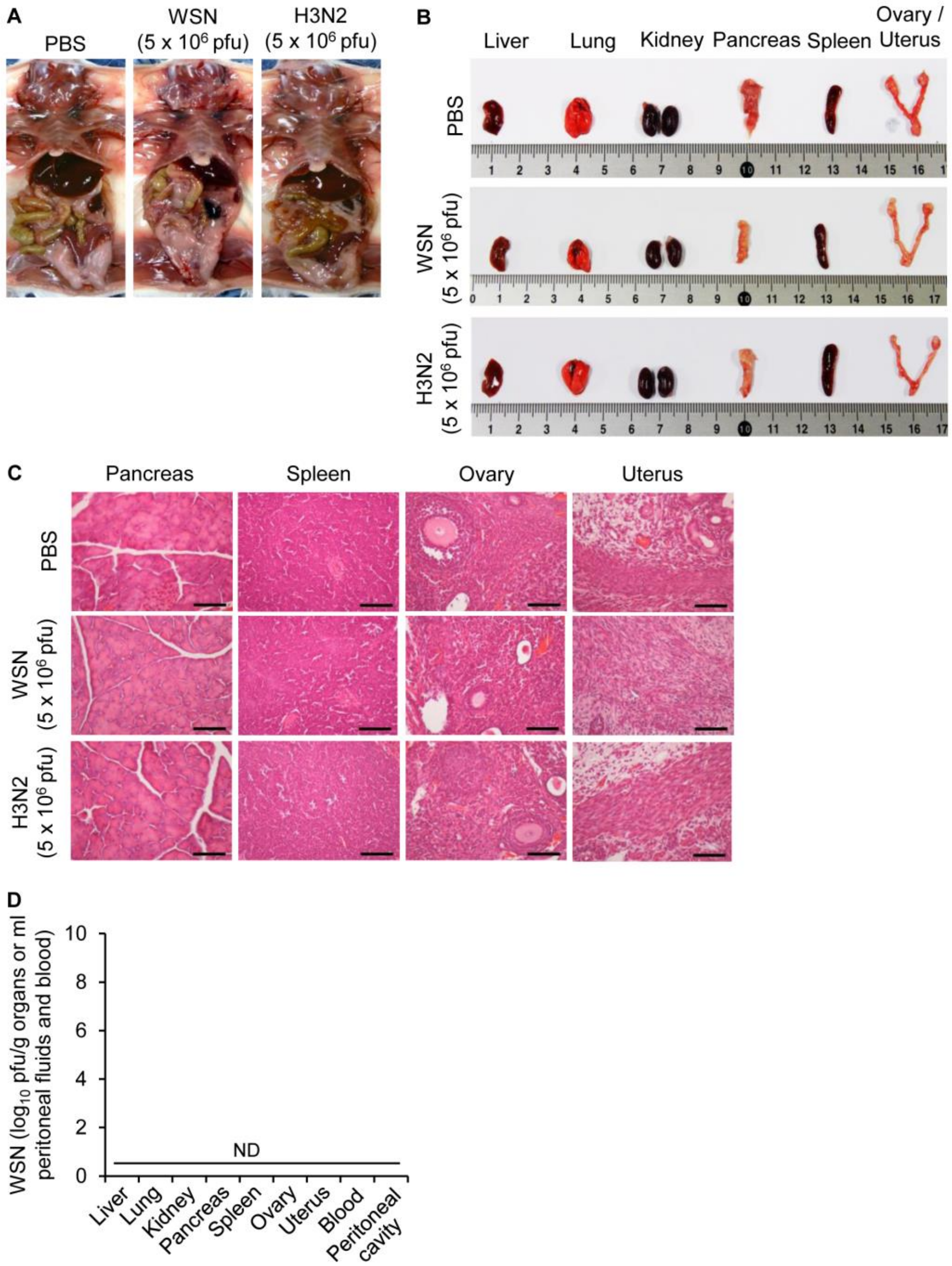

Figure S1. Physical condition of the peritoneal cavity and organs and histopathology of organ tissues after intraperitoneal infection with 5×10^6 pfu influenza A/Hong Kong/4801/2014 virus or influenza A/WSN/1933 virus. BALB/c mice were intraperitoneally infected with PBS, 5×10^6 pfu influenza A/WSN/1933 virus (WSN) or influenza A/Hongkong/4801/2014 virus (H3N2) (n=5/per each group). After 7 days, the mice were sacrificed. (**A**) Images of the exposed peritoneal cavity. (**B**) Organs collected from PBS-treated or virus-infected mice. (**C**) The abdominal organs were excised. Formalin-fixed, paraffin-embedded 5-µm tissue sections of the pancreas, spleen, ovaries, and uterus were subjected to H&E staining. Scale bars, 25 µm. (D) Virus titers in pancreas, uterus, and ovary were determined by plaque assay.

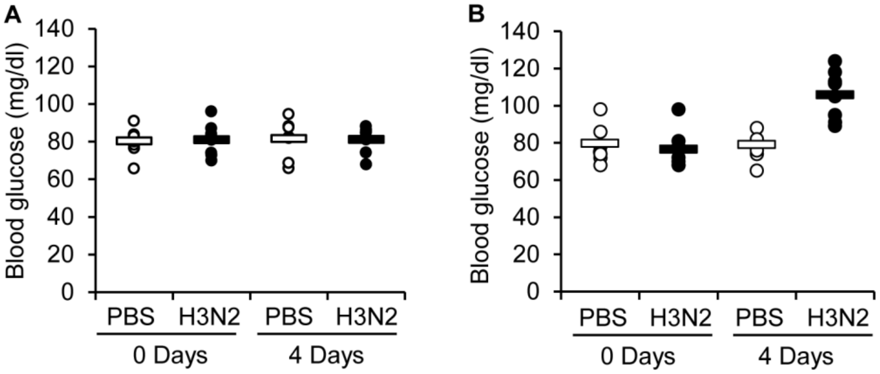

Figure S2. Blood glucose levels in mice infected intraperitoneally with influenza A/Hongkong/4801/2014 virus. BALB/c mice were inoculated intraperitoneally with PBS, 5 × 10^6 pfu (**A**) or 1 × 10^8 pfu (**B**) influenza A/Hongkong/4801/2014 virus (H3N2; n=8/group,). Blood was collected after 12 h of starvation at the starting time point and 4 days after inoculation by retro-orbital puncture. Glucose levels were measured using Standard Mentor NFC.

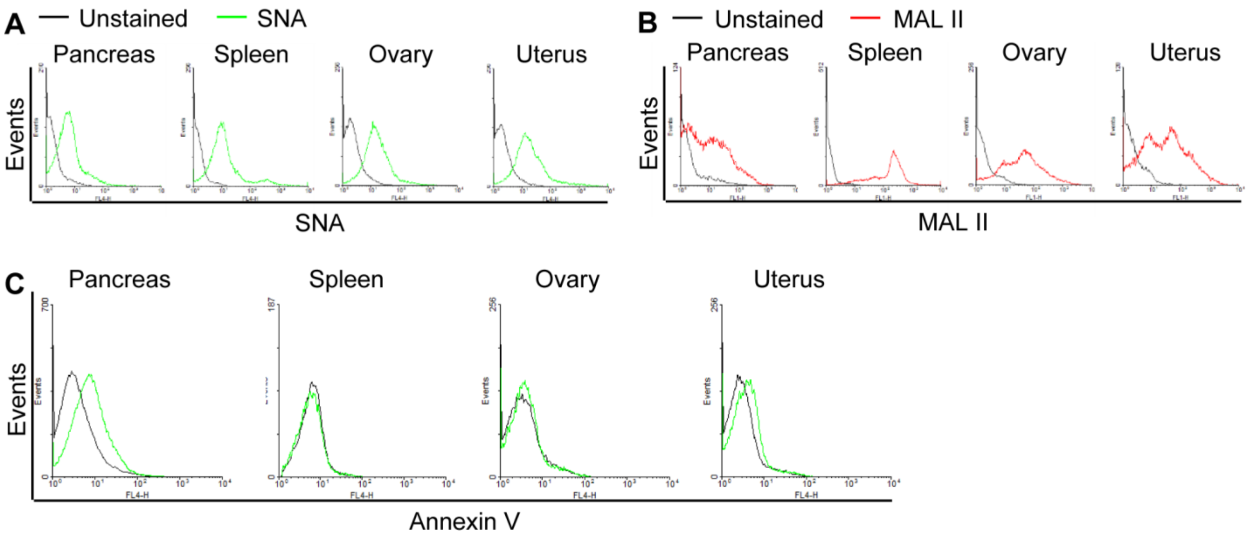

Figure S3. Virus-specific sialic acids in organ tissues and apoptosis of pancreatic cells infected with influenza A/Hongkong/4801/2014 virus. BALB/c mice were sacrificed, and the pancreas, spleen, ovaries, and uterus were excised. Cells from each organ were harvested, stained with fluorescein-conjugated SNA or biotinylated MAL II, and treated with APC-conjugated streptavidin. (**A**, **B**) The binding of SNA (**A**) and MAL II (**B**) to cells from each organ were determined by flow cytometry. (**C**) Cells from each organ were harvested, seeded into 24-well culture plates (1 × 10^6 cells/well), and infected with 1 × 10^6 pfu influenza A/Hongkong/4801/2014 virus. After 72 h of incubation, the cells were collected, stained with APC-conjugated Annexin V, and analyzed by flow cytometry. The numbers of Annexin V-positive cells are shown on the graphs.
